# *Ehrlichia chaffeensis* TRP120-mediated ubiquitination and proteasomal degradation of tumor suppressor FBW7 increases oncoprotein stability and promotes infection

**DOI:** 10.1371/journal.ppat.1008541

**Published:** 2020-04-30

**Authors:** Jennifer Y. Wang, Bing Zhu, LaNisha L. Patterson, Madison R. Rogan, Clayton E. Kibler, Jere W. McBride

**Affiliations:** 1 Department of Pathology, University of Texas Medical Branch, Galveston, Texas, United States of America; 2 Department of Microbiology & Immunology, University of Texas Medical Branch, Galveston, Texas, United States of America; 3 Center for Biodefense and Emerging Infectious Diseases, University of Texas Medical Branch, Galveston, Texas, United States of America; 4 Sealy Institute for Vaccine Sciences, University of Texas Medical Branch, Galveston, Texas, United States of America; 5 Institute for Human Infections and Immunity, University of Texas Medical Branch, Galveston, Texas, United States of America; University of Arkansas for Medical Sciences, UNITED STATES

## Abstract

*Ehrlichia chaffeensis* (*E*. *chaffeensis*) exploits evolutionarily conserved Notch and Wnt host cell signaling pathways to downregulate innate immune host defenses and promote infection. The multifunctional *E*. *chaffeensis* TRP120 effector which has HECT E3 ubiquitin ligase activity, interacts with the host nuclear tumor suppressor F-BOX and WD domain repeating-containing 7 (FBW7). FBW7 is the substrate recognition subunit of the Skp1-cullin-1-FBOX E3 ubiquitin (Ub) ligase complex (SCF) known to negatively regulate a network of oncoproteins (Notch, cyclin E, c-Jun, MCL1 and cMYC). In this study, we demonstrate that TRP120 and FBW7 colocalize strongly in the nucleus by confocal immunofluorescent microscopy and interactions between TRP120 and FBW7 FBOX and WD40 domains were demonstrated by ectopic expression and co-immunoprecipitation. Although *FBW7* gene expression increased during *E*. *chaffeensis* infection, FBW7 levels significantly decreased (>70%) by 72 h post infection. Moreover, an iRNA knockdown of FBW7 coincided with increased *E*. *chaffeensis* infection and levels of Notch intracellular domain (NICD), phosphorylated c-Jun, MCL-1 and cMYC, which are negatively regulated by FBW7. An increase in FBW7 K48 ubiquitination was detected during infection by co-IP, and FBW7 degradation was inhibited in infected cells treated with the proteasomal inhibitor bortezomib. Direct TRP120 ubiquitination of native and recombinant FBW7 was demonstrated *in vitro* and confirmed by ectopic expression of TRP120 HECT Ub ligase catalytic site mutant. This study identifies the tumor suppressor, FBW7, as a TRP120 HECT E3 Ub ligase substrate, and demonstrates that TRP120 ligase activity promotes ehrlichial infection by degrading FBW7 to maintain stability of Notch and other oncoproteins involved in cell survival and apoptosis.

## Author summary

*E*. *chaffeensis* is an obligately intracellular bacterium that replicates in mononuclear phagocytes by secreting effectors that manipulate host cell processes and exploit evolutionarily conserved pathways. This investigation reveals the complex and expanding role of the *E*. *chaffeensis* TRP120 moonlighting effector as a ubiquitin (Ub) ligase targeting host nuclear proteins. Herein, we demonstrate that *E*. *chaffeensis* TRP120 HECT Ub ligase targets the nuclear tumor suppressor Skp1-cullin-1-FBOX E3 ubiquitin (Ub) ligase complex substrate recognition subunit, F-BOX and WD domain repeating-containing 7 (FBW7) for degradation. FBW7 is a central regulator of broadly acting host cell oncoproteins involved in cell proliferation and survival. The reduction in FBW7 through TRP120-mediated ubiquitination increases cellular oncoprotein levels and promotes *E*. *chaffeensis* infection. This study illuminates novel bacterial effector-host interactions, the importance and interplay of both host and bacterial Ub ligases and the Ub-proteasome system for infection, and mechanisms whereby evolutionarily conserved signaling pathways are hijacked by obligately intracellular pathogens.

## Introduction

*Ehrlichia chaffeensis* (*E*. *chaffeensis*) is an obligately intracellular, gram-negative bacterium that exhibits tropism for mononuclear phagocytes and resides in microcolonies within membrane-bound cytoplasmic vacuoles known as morulae [1,2]. *E*. *chaffeensis* survival in the mononuclear phagocyte is dependent in part on pathogen-host interactions involving tandem repeat protein (TRP) effectors that are secreted via the type-1 secretion system and interact with a diverse array of host targets [3–5]. TRPs translocate across the morula membrane via an unknown mechanism and enter the host cell cytosol and nucleus where they function to reprogram the cell through direct interactions with well-defined and lesser known host cell targets [3].

One of the most studied *E*. *chaffeensis* effectors is TRP120, a moonlighting effector that has several defined functions. Early studies demonstrated that surface expressed TRP120 plays a role in host cell entry, but once ehrlichiae are internalized, TRP120 rapidly (<3 h) translocates to the host cell nucleus where it functions as a nucleomodulin, interacts with chromatin-associated proteins and directly binds genes associated with transcriptional regulation, signal transduction and apoptosis [6–9]. TRP120 is also a functional HECT E3 ligase that ubiquitinates host cell substrates including a known interacting partner, polycomb group ring finger protein 5 (PCGF5), a component of the nuclear polycomb repressive complex [10]. TRP120 itself exploits host cell post-translational machinery and is SUMOylated at a canonical motif, which is known to affect TRP120-host target interactions [7,11].

There is a large group of functionally diverse host proteins that interact with TRP120, including FBOX and WD repeat domain-containing 7 (FBW7), the substrate recognition subunit of the eukaryotic Skp1-cullin-1-FBOX E3 ubiquitin (Ub) ligase complex (SCF) [12]. FBW7 regulates a network of well-known oncoproteins (NICD, c-Jun, MCL1, cMYC and cyclin E1) that are involved in cell proliferation, differentiation and regulation of apoptosis through K48 ubiquitination and proteasomal degradation in the nucleus [12,13]. FBW7 contains two primary domains, the FBOX which binds to Skp1 in the SCF complex, and the WD40 domain that recognizes and binds phosphorylated substrates at a conserved Cdc4 phospho-degron motif [13,14]. FBW7 self-regulates through phosphorylation-dependent autoubiquitination, in turn affecting the stability of FBW7 target substrates [15,16]. Furthermore, knockdown of FBW7 leads to a significant enhancement of *E*. *chaffeensis* infection [3]. Although the role of the TRP120-FBW7 interaction in ehrlichial pathobiology remains to be determined, these findings suggest that FBW7 may be a substrate of TRP120 HECT E3 ligase activity.

Recently, our laboratory reported that the *E*. *chaffeensis* TRP120 effector is able to activate Notch signaling in human monocytes, which appears to be critical for establishment and maintenance of intracellular infection [17]. However, the role of Notch signaling, and the mechanisms involved in maintaining Notch activation during infection are not well understood. Notch is an evolutionarily conserved cell signaling pathway that regulates cell proliferation, differentiation and survival [18,19]. Established functions of Notch signaling in the immune systems include regulating B and T cell differentiation, activation of T helper cells, and participation in regulatory functions of T cells [20–24]. Lesser known, but recognized roles of Notch signaling in innate immune system function, include regulation of toll-like receptor (TLR) expression, induction of inflammatory cytokines in response to viral and bacterial infections, and regulation of apoptosis [21,25,26]. However, the exploitation and regulation of Notch signaling pathway by intracellular pathogens has not been described previously.

In this investigation, we have characterized a novel nuclear interaction between the *E*. *chaffeensis* TRP120 effector and the tumor suppressor FBW7. The interaction ultimately enables the *E*. *chaffeensis* TRP120 HECT Ub ligase to engage FBW7 as a substrate to initiate a degradation process that involves K48 ubiquitination and proteasomal degradation. The degradation of FBW7 during ehrlichial infection increases Notch intracellular domain (NICD) levels and stabilizes other prominent FBW7-regulated oncoproteins resulting in ehrlichial infection enhancement. This study provides further insight into how evolutionarily conserved signaling pathways are hijacked by obligately intracellular pathogens.

## Results

### *E*. *chaffeensis* TRP120 interacts with human SCF ligase FBW7

**Fig 1:**
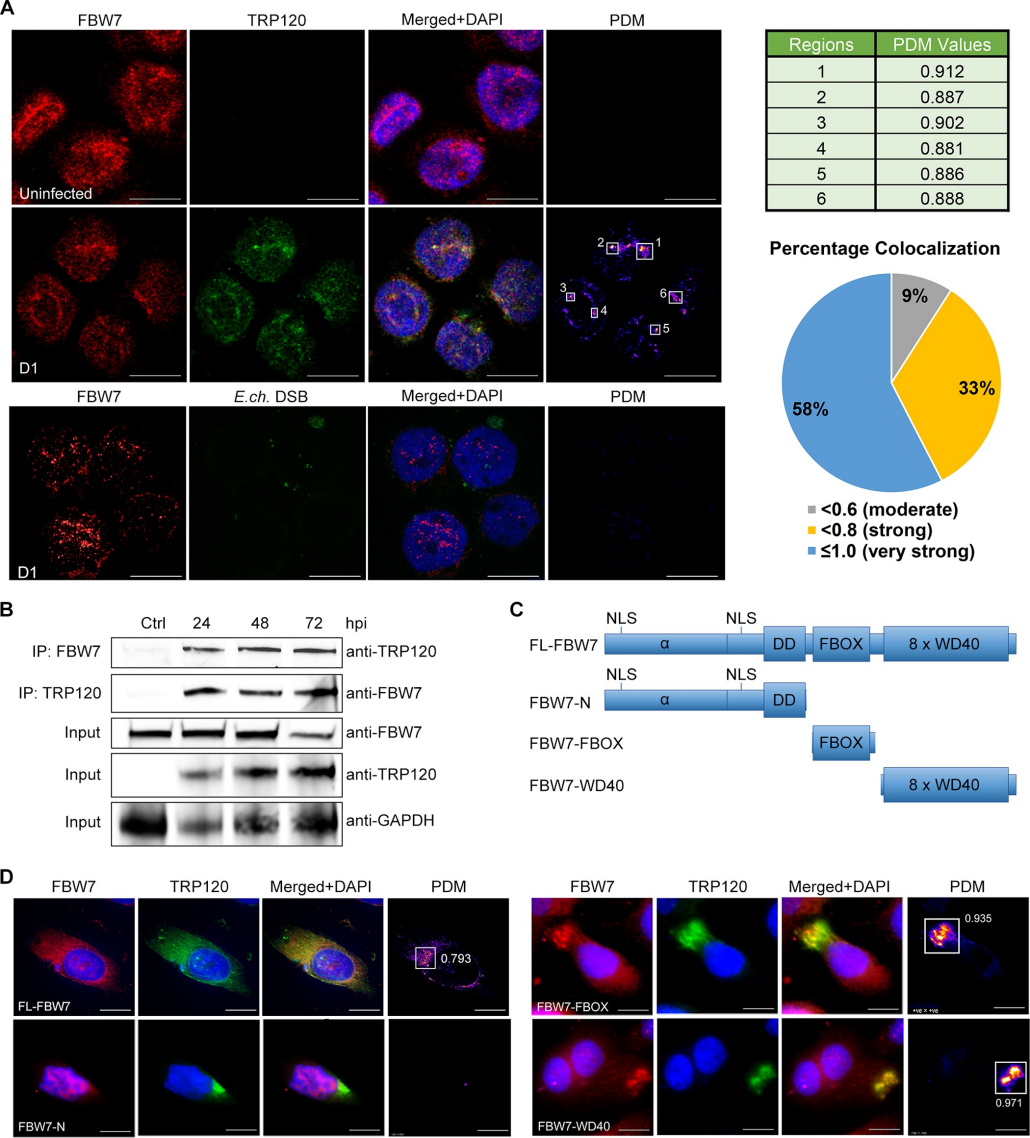
*E*. *chaffeensis* TRP120-FBW7 nuclear colocalization and interaction with FBW7 FBOX and WD40 domains. **A.** Confocal immunofluorescent microscopy demonstrating colocalization of FBW7 (red) and *E*. *chaffeensis* TRP120 (green) in THP-1 cell nuclei at 24 h post-infection (hpi). Nucleus was stained with DAPI (blue). Confocal immunofluorescent microscopy was performed to further demonstrate FBW7 (red) did not colocalize with *E*. *chaffeensis* Dsb (green), suggesting specificity of TRP120-FBW7 nuclear colocalization. Colocalization intensity was determined by Product of the Differences from the Mean (PDM) calculation and shown in the heatmap-like image. The brightest points of colocalization in the PDM image were quantified with Mander’s overlap coefficients (MOC), which ranges between 0 (no colocalization) to 1 (strong colocalization). A table showing MOC values of the highlighted regions of interest shown in the *E*. *chaffeensis*-infected cells (top). In addition, a pie chart was generated from MOC values of multiple regions showing colocalization of TRP120 and FBW7 in the nucleus, where five images (n = 5) containing a total of 33 regions (n = 33) were analyzed, where 58% have very strong colocalization and 33% have strong colocalization. **B.** Co-immunoprecipitation was performed to demonstrate direct interaction between TRP120 and FBW7. THP-1 cells were infected with cell-free *E*. *chaffeensis* and then harvested at 24, 48, and 72 hpi. Anti-TRP120 and anti-FBW7 antibodies were first combined with whole cell lysates for coupling to form antigen-antibody complex. A/G coated magnetic beads were added to the antigen-antibody complex to allow binding and pull down of either TRP120 or FBW7. Western immunoblots were probed with anti-FBW7 or anti-TRP120 (n = 3). **C.** Schematic representation of truncated FBW7 mutant constructs (His-tagged) generated for ectopic expression experiments. **D.** HeLa cells were co-transfected with FL-FBW7 or FBW7 truncated constructs, and TRP120 to examine colocalization by immunofluorescent microscopy. Colocalization was not observed between TRP120 (green) and FBW7-N (red), but strong colocalization between TRP120 and both FBOX and WD40 domains of FBW7 was detected and colocalization strength shown by Mander’s coefficients of 0.935 and 0.971, respectively. This figure was representative of three experiments (n = 3) with technical replicates (n = 2).

Our laboratory reported an interaction between TRP120 and FBW7 with yeast-two hybrid analysis [3,27], but this interaction was not fully investigated or confirmed using other approaches. In order to explore the preliminary yeast two-hybrid results, confocal immunofluorescent microscopy was performed to demonstrate nuclear colocalization of TRP120 with FBW7 in *E*. *chaffeensis-*infected THP-1 cells (Fig 1A). In the control cells, FBW7 was primarily observed in the nuclei and diffusely distributed throughout the cytoplasm. However, in *E*. *chaffeensis-*infected cells, majority of FBW7 was observed in the nuclei in punctate distribution and colocalized with TRP120. Furthermore, intensity correlation analysis was achieved with the Product of the Differences from the Mean (PDM) where the intensities of green and red fluorophores (TRP120 and FBW7, respectively) were calculated. The brightest points of colocalization were also quantified using Mander’s coefficients (0→1, 1 is highest colocalization) with a 58% of total analyzed regions of interest with >0.8, indicating a very strong colocalization correlation between TRP120 and FBW7. We also determined the nuclear colocalization of TRP120 with FBW7 is specific with confocal microscopy demonstrating that FBW7 does not colocalize with *E*. *chaffeensis* Dsb (disulfide bond formation), a protein that is observed in the periplasm of *E*. *chaffeensis* (Fig 1A). In addition, co-IP was used to confirm interaction between TRP120 and FBW7 (Fig 1B). *E*. *chaffeensis-*infected THP-1 cells were harvested at 24, 48 and 72 hpi, and TRP120 and FBW7 were independently immunoprecipitated and detected by Western immunoblot analyses. Using co-IP, we found high levels of FBW7 bound to TRP120 in the infected THP-1 cells compared to uninfected controls. Conversely, we observed high levels of bound TRP120 in FBW7 co-IP samples. Immunofluorescent confocal microscopy, ectopic expression, and co-IP analyses demonstrated colocalization and interaction between TRP120 and FBW7, confirming our previous Y2H results.

### TRP120 interacts with the FBOX and WD40 domains of FBW7

FBW7 contains two major domains required for proper function within the SCF E3 Ub ligase complex: Skp1 binding domain FBOX, and substrate recognition/binding domain WD40 [28]. In order to identify the FBW7 domain interacting with TRP120, dual ectopic expression was performed with TRP120 and FL-FBW7 (Full length FBW7), FBW7-N (N-terminus and dimerization domain), FBW7-FBOX (FBOX domain only), and FBW7-WD40 (WD40 domain with complete C-terminus) (Fig 1C). Co-transfection of TRP120 and the FBW7 truncated constructs was performed in HeLa cells for ectopic expression to visualize colocalization. While TRP120 does not translocate into the nucleus when ectopically expressed, immunofluorescent microscopy revealed FBW7-N was only observed in the nucleus and there was no colocalization observed with TRP120-GFP (Fig 1D). However, based on the DNA sequence alignment obtained from Y2H results, TRP120 was found to bind to both FBOX and WD40 domains of FBW7. This was further supported by *in vitro* pull-down of recombinant TRP120-GST with FBW7 domain constructs (FBOX-His and WD40-His) where both domains were shown to be bound to TRP120 (S1 Fig). In ectopic expression experiments, both FBW7-FBOX and FBW7-WD40 constructs colocalized with TRP120-GFP. Furthermore, the PDM images also illustrated strong colocalization intensity with Mander’s coefficient values of 0.935 and 0.971, indicating majority of TRP120-GFP in the cell colocalized with either FBW7-FBOX or FBW7-WD40. Overall, these findings demonstrate that TRP120 colocalizes specifically with the FBOX and WD40 domains, but not the N-terminus of FBW7.

### FBW7 is degraded during *E*. *chaffeensis* infection

**Fig 2:**
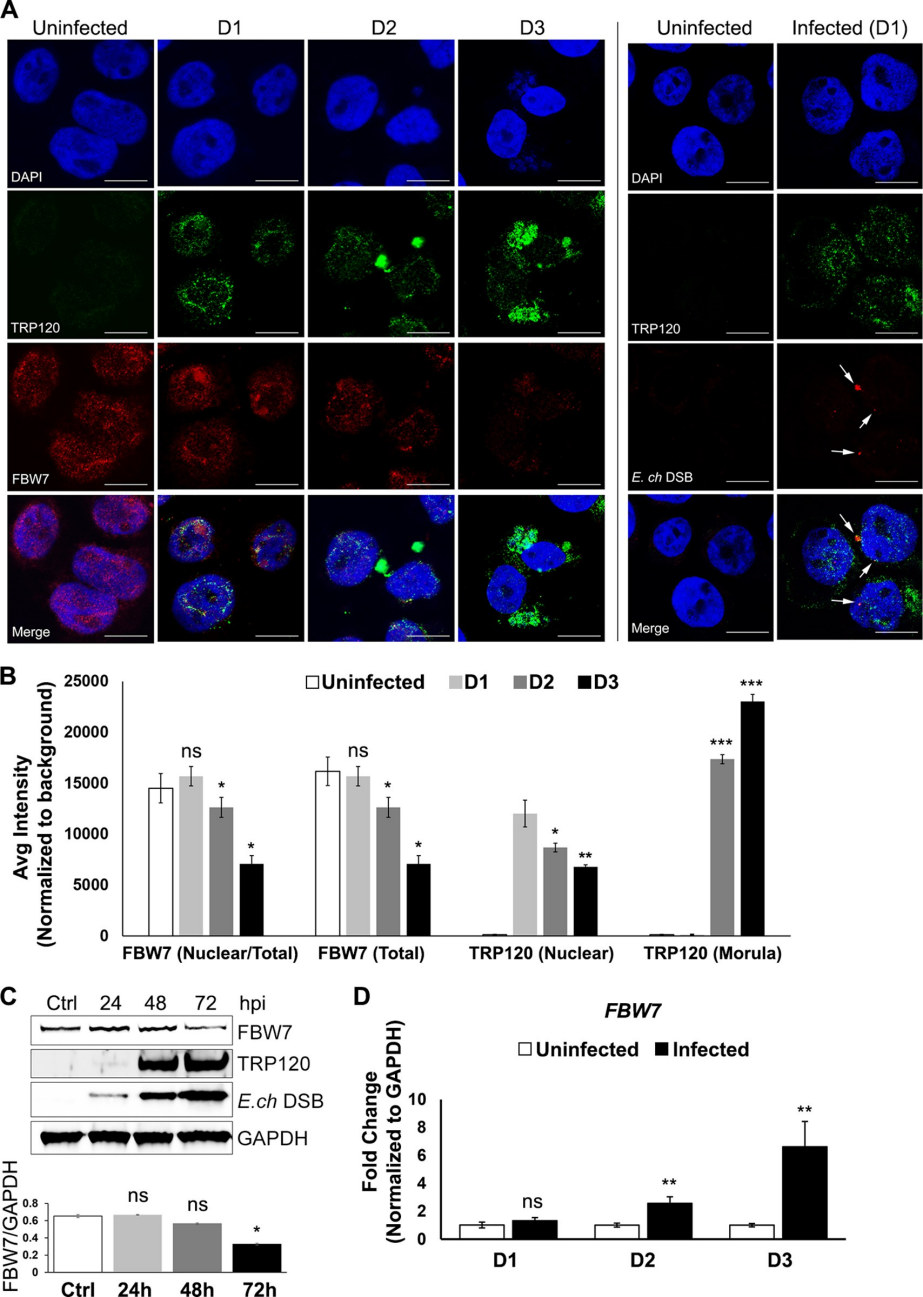
FBW7 is degraded during *E*. *chaffeensis* infection. **A.** Confocal immunofluorescent microscopy was performed to observe colocalization of TRP120 (green) with FBW7 (red) and the temporal changes in FBW7 levels during *E*. *chaffeensis* infection. DAPI was used for nuclear staining (blue). In uninfected cells, FBW7 was primarily observed in the nucleus but was also detectable in the cytoplasm. Day 1 post-infection (pi), FBW7 was detected only in the nucleus and colocalization with TRP120 was observed. By day 2 pi, FBW7 levels decreased ~50% and ~70%, respectively compared to uninfected controls. TRP120 levels decreased in nucleus and increased TRP120 was associated with the morulae 48 and 72 hpi. Confocal immunofluorescent microscopy confirming *E*. *chaffeensis* infection at day 1 pi (presented in the right panel separated by line) with cytoplasmic *E*. *chaffeensis* Dsb (red) detection, also demonstrated nuclear expression of TRP120 (green). For each time point, 3 fields of 30 cells were analyzed (n = 30). **B.** FBW7 and TRP120 fluorescent intensities from the confocal immunofluorescent microscopy (A) were calculated using image J and graphically demonstrated significant nuclear reduction in FBW7 intensity (normalized to total fluorescent intensity) at D2 (*p*<0.05) and D3 pi (*p*<0.05) with presence of nuclear TRP120. A reduction of nuclear TRP120 was also observed at D2 (*p*<0.05) and D3 pi (*p*<0.005) compared to D1 pi, while there was a significant increase of TRP120 in the morulae (*p*<0.0005 for both time points). **C.** Western immunoblots of whole cell lysates harvested from THP-1 cells infected with cell-free *E*. *chaffeensis* for 24, 48 and 72 h with uninfected (ctrl) cells harvested at the same time points identified. Significant decrease of FBW7 level was detected by 72 hpi compared to ctrl (*p*<0.05). n = 4. **D.** Quantitative RT-PCR analysis of *FBW7* expression was performed using cDNA from infected THP-1 cells at 24, 48 and 72 hpi shows a significant increase in *FBW7* expression during infection at 48 and 72 hpi compared to uninfected control (*p*<0.005). GAPDH was used as endogenous control. The analysis was performed from data collected from 3 experiments and technical replicates.

There are several mechanisms for cellular protein regulation, and FBW7 is a well characterized component of the SCF E3 Ub ligase that targets oncoproteins for degradation [14]. However, the molecular mechanisms of FBW7 regulation are not well understood. Recent evidence has shown that FBW7 has multiple upstream regulators such as p53, Pin1, C/EBP-d, Hes-5, Numb, parkin and several microRNAs [29–35]. In order to understand the mechanism of FBW7 regulation during infection, confocal immunofluorescent microscopy was performed on uninfected and *E*. *chaffeensis-*infected THP-1 cells on days 1, 2 and 3 post-infection (Fig 2A). In uninfected THP-1 cells, FBW7 was observed in both nucleus and cytoplasm; however, by day 1 post-infection FBW7 was only observed in the nucleus with TRP120. *E*. *chaffeensis* Dsb staining confirmed *E*. *chaffeensis* infection of THP-1 cells at 24 hpi in which TRP120 was observed in the nucleus but was not detected on ehrlichial morulae. By 48 hpi, TRP120 was primarily observed colocalizing with the morulae but was still present in the nucleus colocalizing with significantly lower levels of FBW7. At 72 hpi, FBW7 nuclear presence was further reduced (>70%) compared to uninfected cells, while TRP120 nuclear level was also reduced (~40%) with significantly increased TRP120 presence on the morulae. The fluorescent intensities of FBW7 and TRP120 from the confocal microscopic images were quantified using image J (Fig 2B), illustrating a temporal decrease in expressions of both nuclear FBW7 and TRP120 levels with significant increased expression of TRP120 on maturing morulae. In addition, FBW7 levels were also quantified using Western immunoblot analysis of whole cell lysates extracted from infected THP-1 cells at 24, 48 and 72 hpi, with uninfected control cells harvested at the same given time points (only a single time point was shown as significant differences were not observed among uninfected cells) (Fig 2C). Consistent with FBW7 visualization by confocal microscopy, there was a gradual reduction in FBW7 levels throughout infection while overall TRP120 levels increased as the infection progressed. But more importantly, nuclear TRP120 remained present throughout infection (Fig 2A and 2B). These results demonstrate that ehrlichial infection and persistent nuclear TRP120 levels coincide with a decrease in FBW7. To investigate FBW7 regulation during *E*. *chaffeensis* infection and the molecular mechanisms involved, qRT-PCR was performed to assess temporal changes in *FBW7* mRNA expression. The results demonstrated progressively increased levels of *FBW7* expression at 24, 48, and 72 hpi (Fig 2D). The decrease in FBW7 protein levels as transcriptional upregulation occurs suggests that FBW7 is post-translationally regulated during infection.

### FBW7 degradation results in increased levels of FBW7 regulated oncoproteins

**Fig 3:**
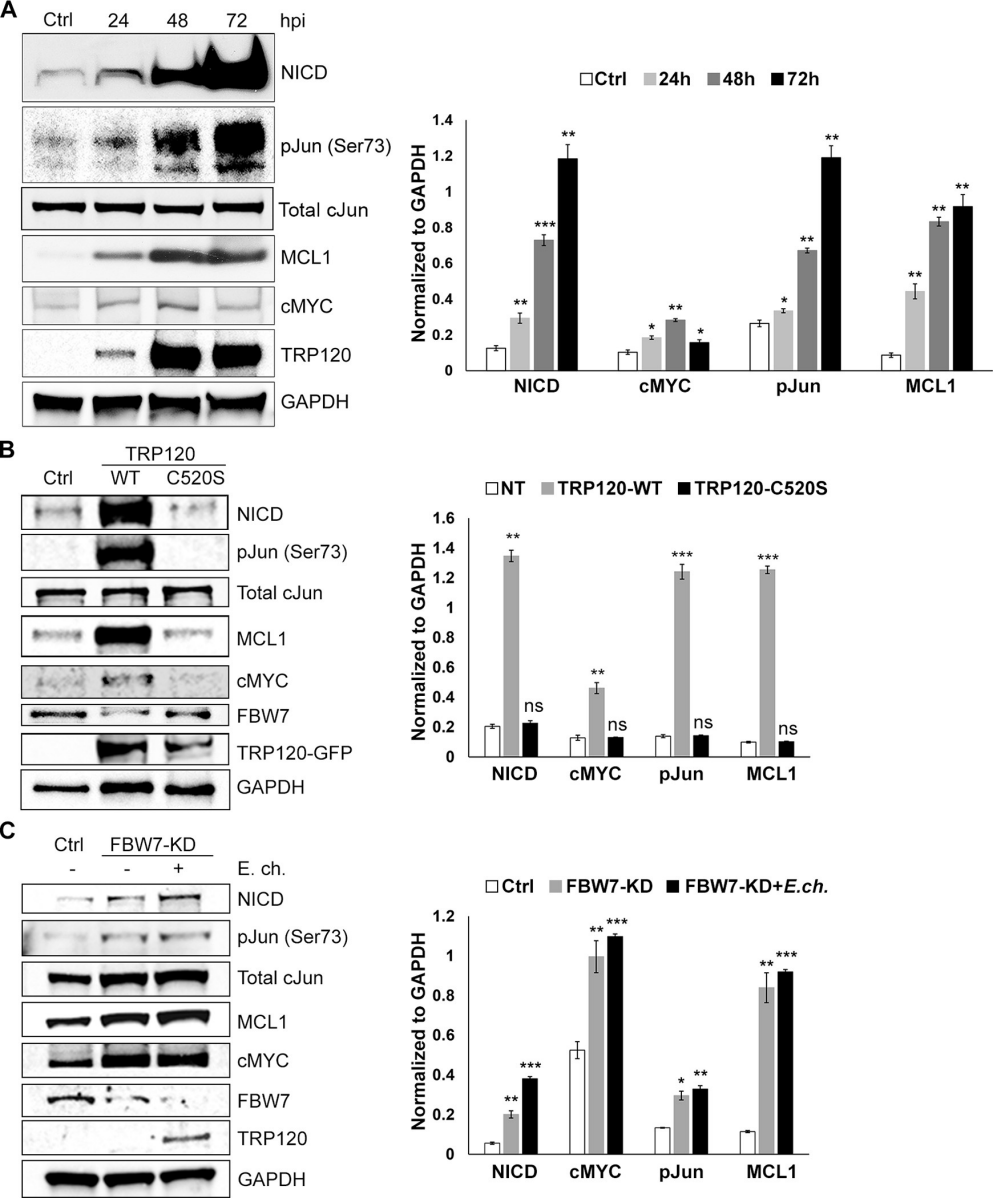
Levels of FBW7-regulated oncoproteins are increased during *E*. *chaffeensis* infection. A. Western immunoblots demonstrating stabilization and upregulation of FBW7 downstream targets: Notch1 intracellular domain (NICD), cMYC, phospho-Jun (p-Jun) and MCL1. Western immunoblots of NICD, cMYC, p-Jun, total c-Jun and MCL1 levels 24, 48 and 72 hpi. GAPDH was used as loading control, and TRP120 to confirm *E*. *chaffeensis* infection (n = 3). Significant temporal increases in NICD, cMYC, p-Jun and MCL1 levels were observed during *E*. *chaffeensis* infection. Densitometry of Western immunoblots (A) was performed to determine levels of each protein using Image J comparing each infected time point to uninfected. Phospho-Jun was additionally normalized to the levels of total c-Jun, then normalized again to the levels of GAPDH (* *p<*0.05, ** *p<*0.005, and *** *p<*0.0005). B. Western immunoblots of uninfected HeLa cells transfected with catalytic-inactive TRP120 (TRP120-C520S) to demonstrate protein levels of NICD, phospho-Jun, MCL1 and cMYC. Densitometry values were measured in ImageJ and plotted (right) to determine the levels of oncoprotein in uninfected cells transfected with TRP120-C520S to be statistically insignificant (ns) compared to control cells (n = 3). C. Western immunoblots demonstrating significant increase of protein levels of NICD, p-Jun, MCL1 and cMYC in both uninfected and infected THP-1 cells with FBW7-KD, compared to uninfected, scrambled siRNA controls (n = 3). Densitometry values were measured in ImageJ and plotted (right), and statistical analysis was calculated for oncoprotein levels in siFBW7 treated cells compared to control (* *p<*0.05, ** *p<*0.005, and *** *p<*0.0005).

In certain cancers, degradation or loss of FBW7 function results in increased levels of FBW7 oncoprotein substrates [12,14]. Notch1 intracellular domain (NICD), c-Jun, MCL1 and cMYC are well characterized and important FBW7-regulated oncoproteins in which overexpression correlates with tumorigenesis [36]. These oncoproteins are also key regulators of cell proliferation, differentiation, and apoptosis [37–39]. Recently, we reported that the Notch signaling pathway is activated during *E*. *chaffeensis* infection by TRP120, resulting in downregulation of TLR2/4 expression [17]. Here, we also observed a significant increase in NICD protein levels (Fig 3A). Activation of the Notch signaling pathway occurs during *E*. *chaffeensis* infection and elevated levels of NICD suggest that pathway activation is maintained in infected cells. Moreover, levels of cellular apoptosis inhibitors phosphorylated c-Jun (p-c-Jun) and MCL1 were also significantly elevated (Fig 3A). While cMYC levels progressively increased at 24 and 48 hpi, levels were significantly reduced at 72 hpi (Fig 3A). Furthermore, we detected increased levels of oncoproteins in TRP120-WT transfected cells compared to cells transfected with catalytic-inactive TRP120 (TRP120-C520S) and cells transfected with control plasmid (Fig 3B). In addition, oncoprotein levels were examined in both FBW7-KD and FBW7-KD with 24 hpi cells and were found to be significantly increased compared to uninfected, scrambled siRNA-transfected control (Fig 3C). These results suggest that TRP120-mediated degradation of FBW7 significantly increases the levels of NICD, p-c-Jun, MCL1 and cMYC.

### FBW7 degradation during infection is K48-Ub-dependent

**Fig 4:**
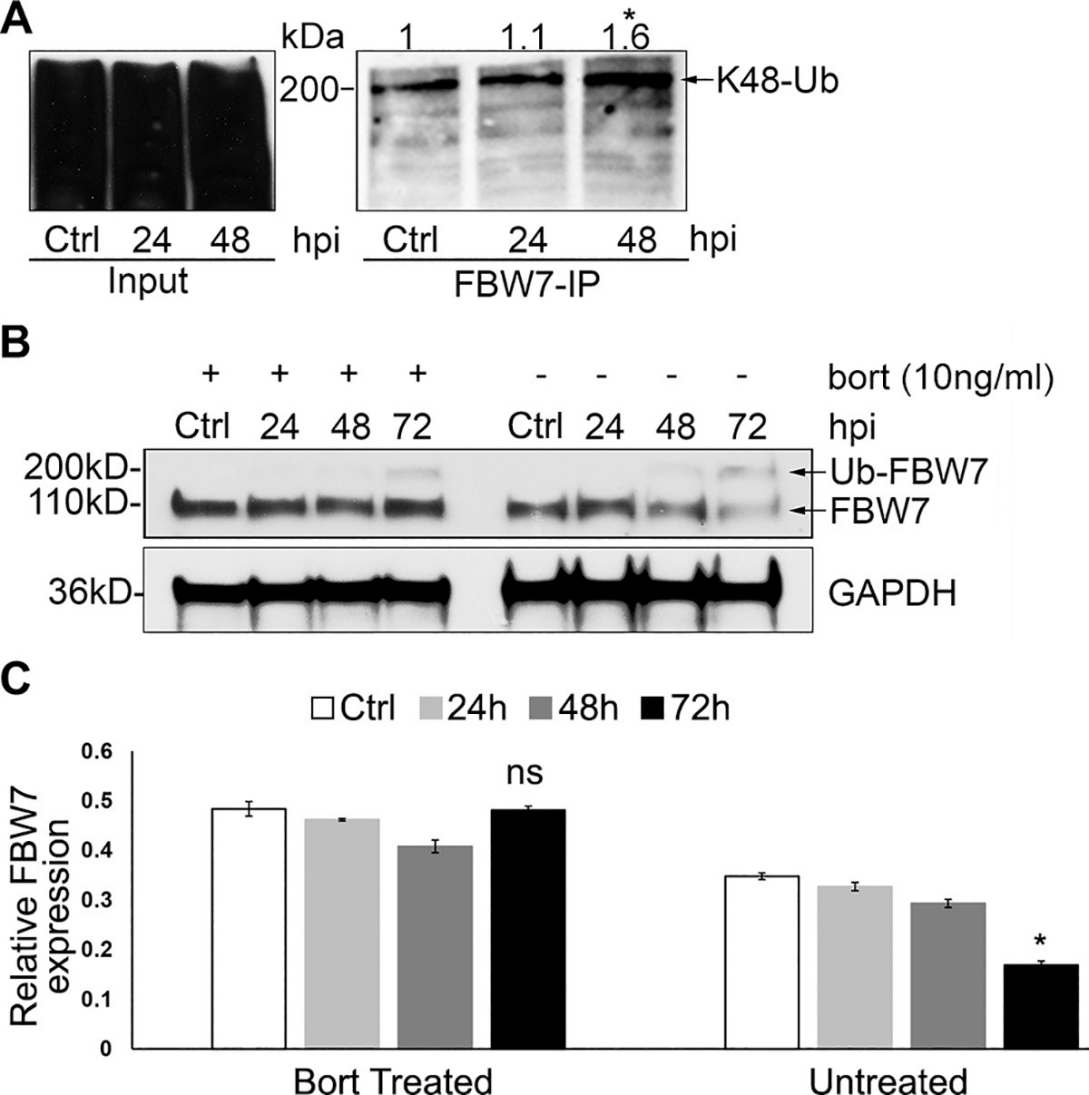
FBW7 is ubiquitinated with K48-Ub and degraded by the proteasome. **A.** Immunoprecipitation of K48-Ub FBW7 from uninfected and *E*. *chaffeensis-*infected THP-1 cells demonstrating increased levels of FBW7 K48-Ub. Lysates were treated with NEM (*N-*ethylmaleimide, equal mass amount to whole cell lysates used) and bortezomib (10ng/ml, 10 h) to prevent deubiquitination and proteasome degradation. Anti-K48-Ub antibody was used to pull down all K48-Ub conjugated proteins. Basal levels of K48-Ub FBW7 at ~200 kDA are observed indicating FBW7 undergoes K48-Ub as a mechanism of protein regulation and turnover. Densitometry values of K48-Ub-FBW7 bands were denoted on top of each band for every time point, and a *p*<0.05 significance comparing 48 hpi to uninfected control (0 hpi) was calculated from three experiments (n = 3). **B.** Western immunoblots demonstrating the effect of bortezomib (bort, 26S proteasome inhibitor) on FBW7 during infection. Whole cell lysates were obtained from both bort-treated (10ng/ml, 10 h) and untreated groups infected with cell-free *E*. *chaffeensis* and uninfected controls at 24, 48 and 72 hpi. Uninfected cells were harvested at the same time points (24, 48 and 72 hpi) with only a single time point (ctrl) shown in the figure as significant differences were not observed among uninfected cells. FBW7 levels remained unchanged during infection in the bort-treated group; however, there was a temporal reduction of FBW7 levels in untreated group, demonstrating FBW7 proteasomal degradation during infection. **C.** Densitometry of Western immunoblots (B) performed using image J. Statistical analysis was done by comparing data from infected cells to control cells at respective time points (n = 3). A p<0.05 significance was determined for FBW7 level at 72 hpi in the untreated group compared to uninfected control in the same group.

While post-translational regulation of FBW7 is not well studied, there is evidence demonstrating that autoubiquitination is the primary mechanism for self-regulation and turnover [40]. In the absence of downstream substrates, FBW7 is ubiquitinated within the SCF complex by an autocatalytic reaction, which leads to its proteasomal degradation [41]. Other studies have also reported that in neurons, parkin promotes FBW7β ubiquitination-dependent degradation; however, the exact mechanism was not determined [32]. For a protein to be targeted by the 26S proteasome, it must be ubiquitinated with a polyubiquitin chain such as K48-Ub. To determine whether FBW7 was ubiquitinated with K48-Ub, co-IP was performed to examine the ubiquitination status of FBW7 during ehrlichial infection (Fig 4A). FBW7 was immunoprecipitated from *E*. *chaffeensis-*infected and uninfected cells harvested at 24 and 48 hpi and treated with NEM and bortezomib to inhibit deubiquitination and degradation. The immunoblots revealed a constitutive level of ubiquitinated FBW7 (Ub-FBW7, 200 kDa) in uninfected control cells, which is consistent with maintenance of FBW7 levels by K48 autoubiquitination. In infected cells, K48-ubiquitinated FBW7 levels increased in bortezomib-treated cells. Moreover, decreased FBW7 was observed at 24, 48 and 72 hpi in untreated cells, but was stable throughout infection in bortezomib-treated (10ng/ml, 10 h) cells (Fig 4B and 4C). These results further suggest that increased FBW7 degradation during *E*. *chaffeensis* infection occurs through K48 ubiquitination and is proteasome-dependent.

### TRP120 ubiquitinates FBW7 for degradation

**Fig 5:**
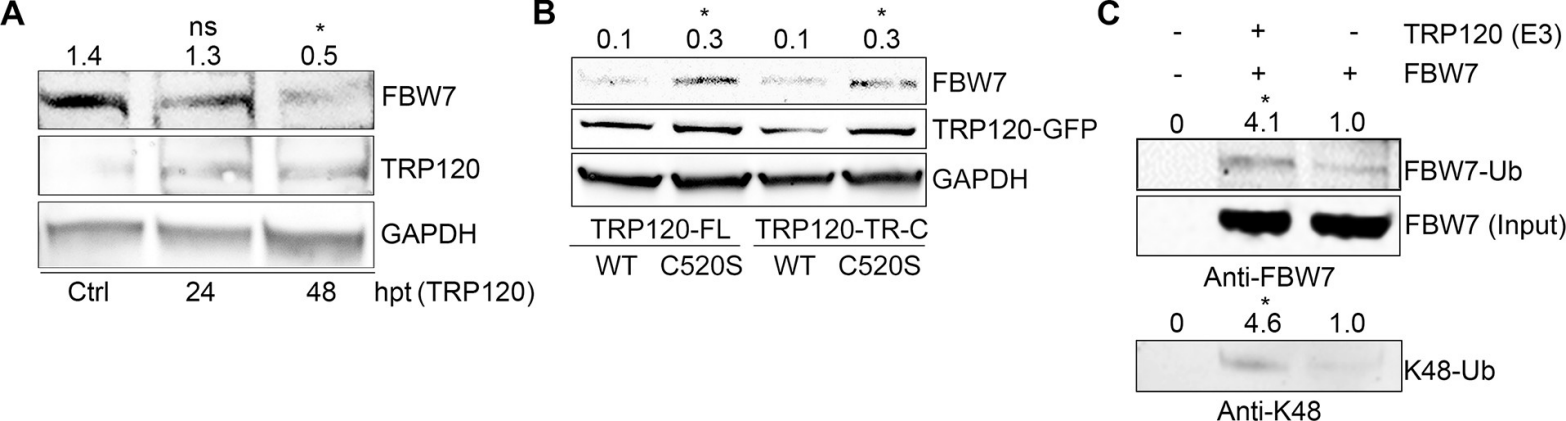
*E*. *chaffeensis* TRP120 HECT E3 Ub ligase activity mediates K48 ubiquitination and degradation of FBW7. **A.** Levels of FBW7 decrease in HeLa cells ectopically expressing TRP120-GFP at 0, 24, and 48 hours post transfection (hpt). Densitometry values of FBW7 for each time point were denoted above respective bands (ctrl: 1.4 ±0.03; 24 hpt: 1.3±0.08; 48 hpt: 0.5±0.09), with Significantly (*p*<0.05) decreased levels of FBW7 were detected at 48 hpt with TRP120 compared to control (n = 3). **B.** HeLa cells were transfected with TRP120 WT and HECT E3 ligase catalytic mutants (TRP120-FL-C520S and TRP120-TR-C-C520S). Increased degradation of endogenous FBW7 was detected in TRP120-WT compared TRP120-C520S mutants lacking Ub ligase function. Densitometry values of FBW7 for each sample group were labeled above respective bands (TRP120-FL-WT: 0.1±0.02; TRP120-FL-C520S: 0.3±0.04; TRP120-TR-C-WT: 0.1±0.004; TRP120-TR-C-C520S: 0.3±0.03), with *p*<0.05 significance for both TRP120 transfection groups (TRP120-FL and TRP120-TR-C) comparing levels of FBW7 in cells transfected with catalytic-inactive TRP120 mutant (C520S) to wildtype TRP120 (WT) (n = 3). **C.** *In vitro* ubiquitination of native FBW7 by *E*. *chaffeensis* rTRP120 using anti-FBW7 and anti-K48 antibodies for detection. Increased FBW-Ub was detected at ~200 kDa in the presence of rTRP120 compared to controls without TRP120. K48 Ub was also detected on FBW7-Ub in the presence of rTRP120 indicating TRP120 directly ubiquitinates FBW7 with K48-Ub chains. Densitometry values of both FBW7-Ub (FBW7+TRP120: 4.1±0.17; FBW7-TRP120: 1.0±0.02) and K48-Ub (FBW7+TRP120: 4.6±0.06; FBW7-TRP120: 1.0±0.01) were denoted above each bands, with *p*<0.05 significance comparing levels of either FBW7-Ub or K48-Ub in the presence of rTRP120 to the absence of rTRP120 as E3 ligase (n = 4).

TRP120 has HECT E3 ligase activity and one host cell substrate (PCGF5) has been identified [10]. Thus, we considered that FBW7 could also be a TRP120 substrate. To examine this question, we transfected TRP120-GFP in HeLa cells to determine if ectopically expressed TRP120 affected FBW7 stability. Indeed, when HeLa cells were transfected with TRP120-GFP plasmid for 24 and 48 h, there was a progressive reduction of FBW7 levels compared to controls (Fig 5A). These results indicate that TRP120 does impact FBW7 stability. We then compared FBW7 stability in HeLa cells transfected with TRP120-WT and TRP120-C520S, a mutant TRP120 lacking the E3 ligase function [10]. FBW7 stability was increased in cells expressing TRP120-C520S compared to TRP120-WT. In addition, cells expressing TRP120-TR-C-C520S, a truncated TRP120 containing only the TR domains and mutant C-terminus HECT E3 ligase catalytic domain, also resulted in stability of FBW7 further demonstrating FBW7 is a TRP120 substrate (Fig 5B). To confirm that FBW7 is a TRP120 substrate, *in vitro* ubiquitination assays were performed to demonstrate TRP120 directly ubiquitinates FBW7. The *In vitro* ubiquitination experiment revealed that in the presence of rTRP120, Ub-FBW7 (~200 kD) was detected at higher levels compared controls. Similarly, increased K48-Ub was detected on FBW7 in the presence of rTRP120 (Fig 5C), which indicates that TRP120 ubiquitinates FBW7 with K48-Ub. Furthermore, the elimination of TRP120 N-terminus did not affect TRP120 Ub ligase activity and FBW7 degradation, demonstrating that the TRP120 N-terminal domain has no role in the interaction or in TRP120 Ub ligase activity. Collectively, these results demonstrate that FBW7 is a substrate of TRP120 Ub ligase activity [10], resulting in FBW7 proteasomal degradation.

### Changes in FBW7 levels impact *E*. *chaffeensis* infection

**Fig 6:**
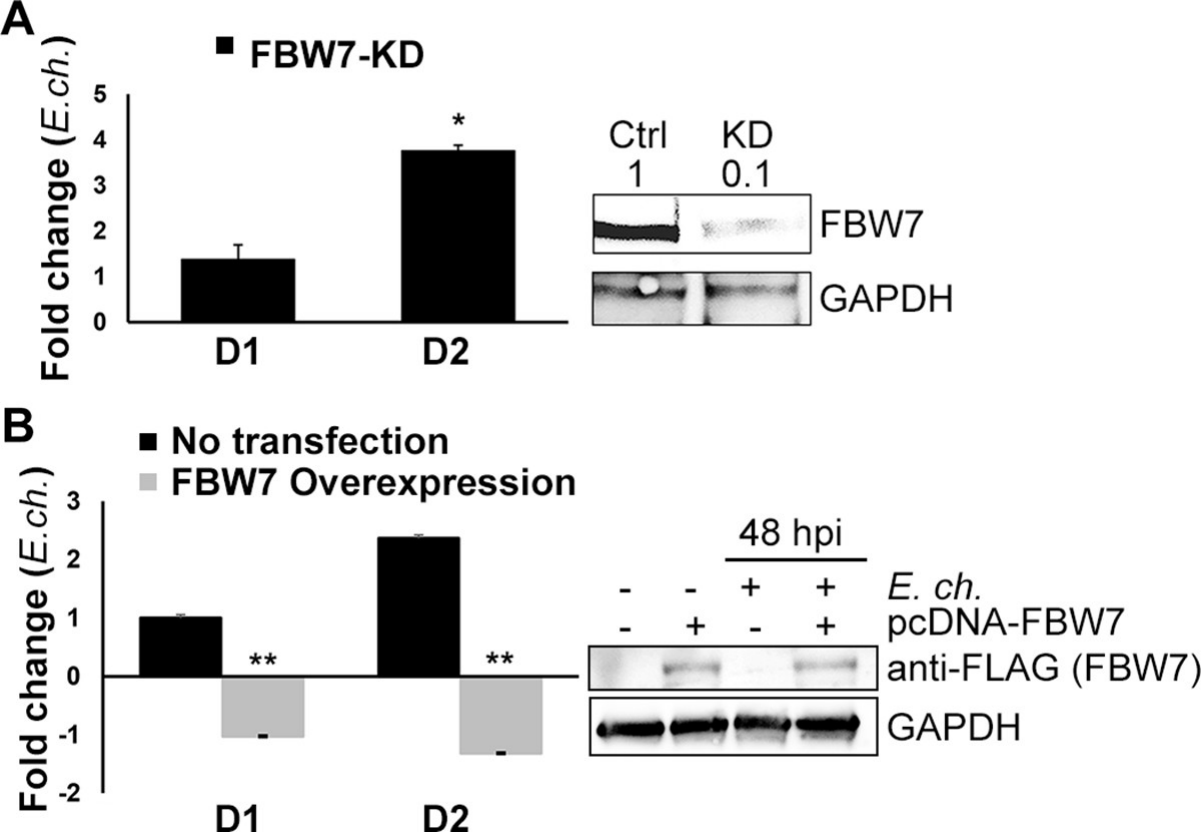
Changes in FBW7 levels impact *E*. *chaffeensis* infection. **A.** iRNA knockdown of FBW7 in *E*. *chaffeensis-*infected THP-1 cells at 24 and 48 hpi. Scrambled siRNA was used as RNAi control. Western immunoblot (right) demonstrated ~90% knockdown (KD) of *FBW7*, densitometry values were labeled above each band. Cells were harvested for qPCR to assess changes in *E*. *chaffeensis* infection. For all the data points from both uninfected and infected KD cells, the data was first normalized to cells transfected scrambled siRNA (infected and uninfected, respectively). Then, the infected group was normalized to uninfected group to determine the fold change. A significant increase (3-fold) in *E*. *chaffeensis* load was detected in FBW7-KD cells (*p≤*0.05). **B.** Overexpression of FBW7 was achieved by transfecting HeLa cells with pcDNA3.1+/C-(K) DYK-FBW7 (denoted as pcDNA-FBW7 in the figure), and negatively impacted *E*. *chaffeensis* infection (*p≤*0.005) in infected HeLa cells. Quantitative PCR analysis was performed similarly to panel A. Western Immunoblot (right) confirmed ectopic overexpression of FBW7 and quantitated with densitometry values as denoted above each band. For both panels A and B, the data shown is representative of 3 experiments (n = 3) with technical replicates (n = 3).

We examined the effect of FBW7 on *E*. *chaffeensis* infection using siRNA knockdown of FBW7 in THP-1 cells. *E*. *chaffeensis* infection was significantly and progressively increased by 50% by 48 hpi in FBW7-KD cells compared to control transfected with scrambled siRNA (Fig 6A). Conversely, when FBW7 was overexpressed in infected HeLa cells, *E*. *chaffeensis* infection was reduced up to 3-fold compared to infected control cells (Fig 6B). Collectively, these results further support the conclusion that FBW7 stability plays a crucial role in promoting cell survival and *E*. *chaffeensis* infection in monocytes.

## Discussion

Recent investigations of *E*. *chaffeensis* pathobiology have revealed how TRP effectors exploit host signaling and post translational pathways and other cellular processes in order to evade host innate immune mechanisms and establish intracellular infection [6,9,42–44]. Specifically, *E*. *chaffeensis* has evolved mechanisms to avoid recognition by innate immune receptors and destruction in the phagolysosome, inhibit apoptosis, and alter cellular signaling required for host cell defense function [45]. Recently, we reported that *E*. *chaffeensis* exploits evolutionarily conserved host cell signaling pathways such as Wnt and Notch to modulate innate host defenses to promote infection in monocytes [5,17]. Both Wnt and Notch are known for their role in regulating cellular proliferation and cell fate, but they have also been shown to influence innate immune defenses such as apoptosis and autophagy [46,47], and previous studies have determined the Notch signaling regulates TLR expression [48,49]. Notably, we have recently determined that *E*. *chaffeensis* exploitation of Notch signaling downregulates TLR2/4 expression during infection [17].

In a previous study, we reported that FBW7, a negative regulator of intracellular Notch, interacted with TRP120 [3]. Three FBW7 isoforms with similar function occupy distinct subcellular compartments. With regards to the present investigation, FBW7α (referred as FBW7 in this study) is the most abundantly expressed in many cell types, including monocytes. It is primarily found in the nucleus due to the presence of two canonical NLS sequence motifs where it functions as the substrate recognition subunit of the SCF E3 ligase complex [13,15,41]. Studies have shown that FBW7 negatively regulates downstream substrates strictly in the nucleus through K48 ubiquitination to facilitate proteasomal degradation [36,50–53]. However, when FBW7 is displaced from the nucleus through deletion of the NLS sequence motifs, the SCF complex becomes unstable and unable to ubiquitinate downstream substrates [54,55].

TRP120 has been shown to translocate to the host cell nucleus where it binds host genes associated with transcriptional regulation, signal transduction and apoptosis [3]. Thus, we explored the molecular details of the interaction between *E*. *chaffeensis* TRP120 and FBW7 in the host cell nucleus and determined the fate and regulatory mechanism of FBW7 as a result of this interaction. Strong colocalization was observed with confocal microscopy between TRP120 and FBW7 primarily in the nucleus at early time points during infection, which is consistent with the temporal/spatial dynamics of TRP120 nuclear translocation and function. Furthermore, ectopic expression results revealed that TRP120 colocalizes with adjacent FBOX and WD40 domains of FBW7, suggesting the interaction with these domains may facilitate ubiquitination of lysine residues within FBW7.

Under normal cellular conditions, FBW7 regulation occurs at both transcriptional and post-translational levels [41]. Transcriptional regulation of FBW7 includes CCAAT/enhancer-binding protein delta (CEPBδ), and it has shown to directly inhibit *FBW7* expression; Hes5, a downstream target of Notch signaling, has also been reported to repress FBW7 expression [34,56–58]. Other studies have reported that miRNA-27a, miRNA-223 and miRNA-25 inhibit the expression of *FBW7* to promote tumorigenesis [59–62]. However, the main mechanism involved in FBW7 turnover appears to be autoubiquitination [41]. In the absence of substrates, autoubiquitination of FBW7 occurs within the SCF complex, and is dependent on phosphorylation priming at Ser227 then facilitated by the peptidyl-prolyl *cis/trans* isomerase Pin1 [29]. Another study demonstrated that E3 ligase parkin ubiquitinates FBW7β in the neurons; however, the exact molecular mechanism was not determined [32]. In the context of *E*. *chaffeensis* infection of monocytes, we have determined that FBW7 is progressively degraded and the level of FBW7 is not restored at any time during infection despite upregulated *FBW7* gene expression. Further investigation demonstrated that the decrease in FBW7 is independent of transcriptional regulation. Instead, FBW7 levels begin to decrease after TRP120 translocation into the nucleus and progressively decrease during infection. This finding suggests that TRP120 negatively regulates FBW7 in order to stabilize downstream oncoproteins involved in regulation of cell proliferation and survival, thereby impacting *E*. *chaffeensis* infection.

*E*. *chaffeensis* survival is dependent on activation of cell signaling pathways, such as Wnt and Notch to regulate cellular proliferation, differentiation and innate host defenses including autophagy and apoptosis [46,47]. Despite the well characterized role of the Notch pathway in cell differentiation, less is known regarding its role in regulating innate immunity. However, accumulating evidence has shown a complex role of Notch signaling pathway in innate immunity regulation [48,49,63]. During *E*. *chaffeensis* infection, we have determined that TRP120 not only acts as nucleomodulin to upregulate *NOTCH1* transcription, but is also functions as Notch ligand mimic to activate Notch signaling [5,9,17]. Moreover, as demonstrated in this investigation, TRP120 E3 ligase activity is involved in regulating Notch signaling by degrading the negative regulator FBW7 to maintain Notch signaling. These findings not only reveal the importance of Notch signaling pathway in promoting infection, but also highlight the unique moonlighting functions of TRP120. Notably, this is the first bacterial effector shown to exhibit such a diversity of functions in different cellular contexts during infection including direct activation and regulation of the same signaling pathway.

The TRP120-FBW7 interaction results in stabilization of other well-known oncoproteins. As occurs in tumor cells, the reduction of FBW7 leads to increased levels of anti-apoptotic proteins p-c-Jun, MCL1 and cMYC during ehrlichial infection. Oncoprotein c-Jun is part of the JNK signaling pathway that plays a vital role in apoptosis, inflammation, cytokine production and metabolism [64]. Through a series of phosphorylation events, c-Jun is phosphorylated and activated for downstream AP-1 gene transcription that contribute to a diverse regulatory mechanism involved in cell proliferation, cell differentiation and apoptosis [65]. However, like tumor cells, elevated transcription factor p-c-Jun is observed as response to the degradation of FBW7, and we have also shown direct *E*. *chaffeensis* TRP32 nucleomodulin-mediated upregulation of *JUN* expression during infection [66]. Like Notch, both upregulation of mRNA expression and elevation of protein levels suggests the stabilization of c-Jun activity is crucial for *E*. *chaffeensis* intracellular survival. This is also complemented by the increase in MCL1 protein levels, an anti-apoptotic member of the BCL-2 family known to block the release of cytochrome *c* that signals for cell death [67,68]. Although the oncoproteins regulated by FBW7 increased, cMYC does not exhibit the same temporal levels we noted with NICD, p-c-Jun and MCL1. Instead, the decrease in cMYC level at 72 hpi correlates with the downregulation in *MYC* expression reported during infection. The proportional reduction in *MYC* expression suggests a negative feedback mechanism to modulate cMYC levels in the cells that eventually overwhelms the accumulation of cMYC from the loss of FBW7 [5,69]. Finally, we have revealed that TRP120-FBW7 interaction regulates downstream oncoproteins NICD, cMYC, c-Jun and MCL1, which appear to play essential roles in delaying host cell apoptosis and further studies are required to fully understand their roles in promoting *E*. *chaffeensis* infection.

Several bacterial effectors are known to target host proteins to manipulate the host ubiquitination system, such as *Shigella* OspI and OspG which interact with host E2 proteins. In addition, *Salmonella* SopA has HECT E3 ligase activity that has been suggested to play a regulatory role in host ubiquitination pathways [70–74]. A recent study reported that *Salmonella* SopA enhances ubiquitination of host protein TRIM65 to modulate innate immune responses by inducing interferon-β expression [75]. However, other specific host substrates that interact with OspI, OspG or SopA have not been identified. Notably, a TRP120 HECT ligase substrate (PCGF5) has been identified [10]. The interaction between TRP120 and PCGF5 has been shown to occur in the nucleus during early infection (24 h) and with cytoplasmic morulae during late infection (72 h) [6]; however, TRP120 appears to interact with FBW7 in the nucleus throughout the course of infection resulting in Ub-mediated degradation of FBW7.

We have previously demonstrated TRP120 Ub ligase function and identified PCGFs as host substrates [6,10]. This study identifies another TRP120 Ub ligase substrate and further demonstrates the role of TRP120 in regulating the levels of specific host cell proteins that are known to interact with TRP120. We demonstrated that TRP120 targets FBW7 with K48-Ub linkage chain, which is one of the major post translational modification events leading to proteasomal degradation [76]. In addition, K48 ubiquitination of FBW7 occurs through the functional TR and C-terminal domains of TRP120 and does not require the N-terminus. This is consistent with previous reports that have demonstrated the TR domain of TRP120 is important for interactions with host proteins, and the C-terminal domain has conserved HECT ligase catalytic domain [3,4,10]. Notably, a major finding of this investigation is the identification of FBW7 as the second TRP120 E3 ligase substrate. Notably, *E*. *chaffeensis* is the only pathogen to our knowledge known to target FBW7 during infection.

The TRP120-FBW7 interaction reveals that the TRP120-mediated ubiquitination-dependent degradation of FBW7 to be critical for *E*. *chaffeensis* infection. We have previously shown that the reductions in FBW7 through iRNA knockdown leads to enhanced ehrlichial infection [27]. In this study, we have determined that degradation of FBW7 by TRP120 E3 ligase activity is a mechanism to reduce FBW7 levels. In addition, reintroduction of FBW7 most likely inhibits and corrects downstream pro-survival NICD, c-Jun, MCL1 and possibly cMYC cell signaling to restore monocyte apoptosis, thus preventing cell survival and propagation of *E*. *chaffeensis* infection. This strongly supports the importance of TRP120-mediated degradation of FBW7 in the maintenance of Notch signaling as a mechanism to prevent apoptosis to promote *E*. *chaffeensis* infection.

**Fig 7:**
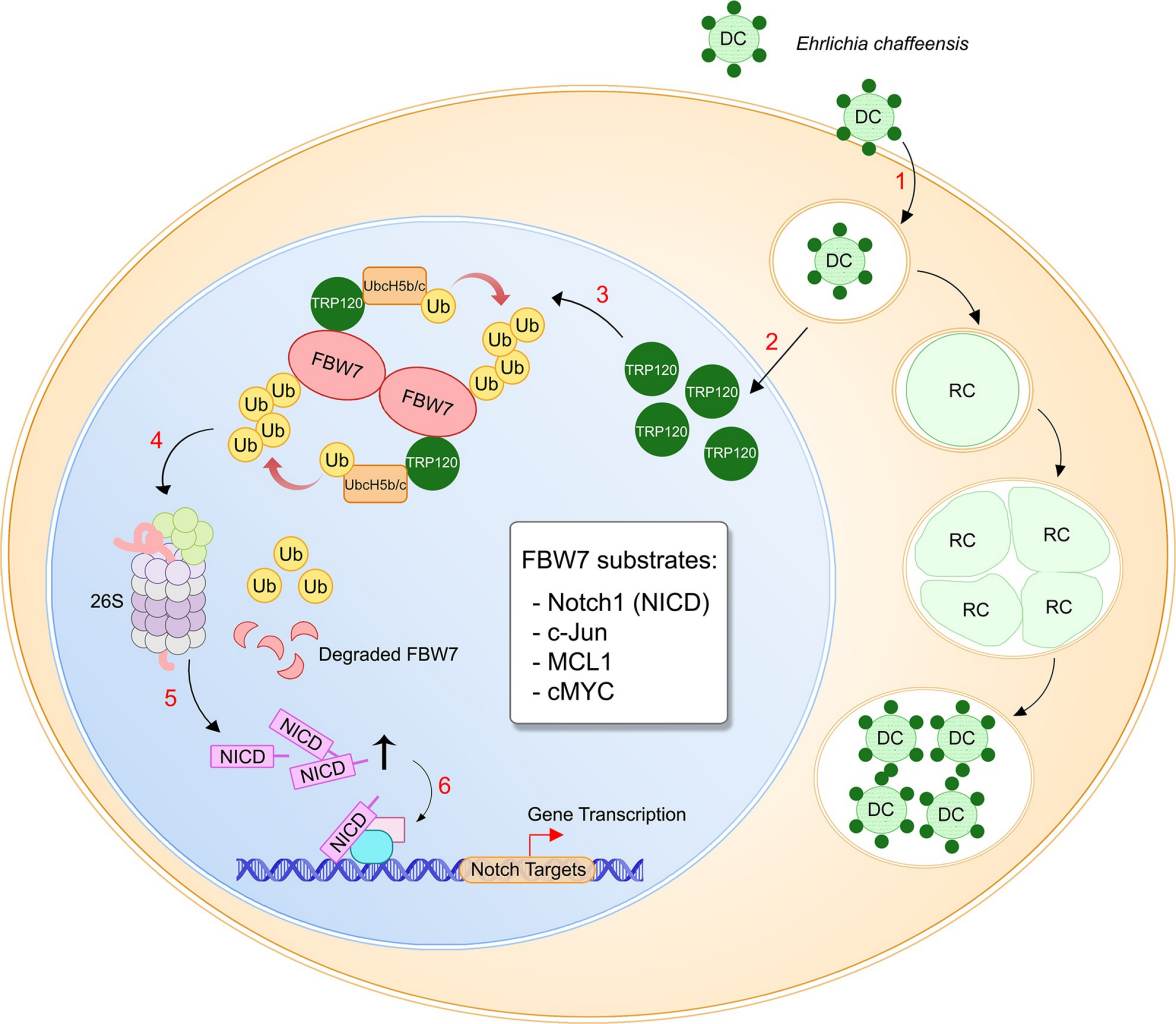
Proposed model of *E*. *chaffeensis* TRP120 HECT E3 ligase regulation of FBW7 during infection. (1) Infectious dense-cored *E*. *chaffeensis* expressing TRP120 on the surface enter host monocytes through receptor-mediated phagocytosis and replicates in a cytoplasmic vacuoles that do not fuse with lysosomes; (2) TRP120 is secreted via type-1 secretion system into host cell within 3 h of entry and translocates into the host nucleus; (3) TRP120 binds to phosphorylated FBW7 homodimer in *trans* conformation and ubiquitinates with K48-Ub chains; (4) FBW7-K48-Ub is then degraded by the 26S proteasome, presumably in the nucleus; (5) TRP120-mediated degradation of FBW7 leads to stabilization and accumulation of downstream oncoprotein targets including NICD, c-Jun, MCL1 and cMYC; (6) Pro-survival oncoproteins activate downstream gene transcription to delay host cell apoptosis to promote *E*. *chaffeensis* infection.

Through this investigation, we have gained an understanding of the importance of TRP120 interaction with FBW7 during *E*. *chaffeensis* infection. An overview of this interaction and downstream effects are illustrated by the proposed model (Fig 7) that can be summarized as follows: 1) *E*. *chaffeensis* attaches and enters a monocyte as dense-core ehrlichiae where TRP120 is expressed on the bacterium surface; 2) TRP120 is secreted via type-1 secretion system, and translocates to the host cell nucleus via an unknown mechanism; 3) TRP120 ubiquitinates FBW7 in the nucleus; 4) K48-Ub-FBW7 is then degraded by the 26S proteasome most likely in the nucleus; 5) degradation of FBW7 results in increased levels of downstream pro-survival markers (NICD, p-c-Jun, MCL1 and cMYC) resulting in the maintenance of Notch signaling activation and anti-apoptotic programming; 6) finally, the pro-survival markers activate downstream gene expression to suppress host innate immunity and delay monocyte apoptosis, thus creating a favorable environment for *E*. *chaffeensis* replication.

## Materials and methods

### Cell culture

Human monocytic leukemia cells (THP-1) were grown and maintained in RPMI medium 1640 with L-glutamine and 25 mM HEPES buffer (Invitrogen, Carlsbad, CA), supplemented with 10% fetal bovine serum (HyClone, Logan, UT). Henrietta Lack’s cervical epithelial adenocarcinoma (HeLa) cells were propagated in MEM medium with Earle’s Salts and L-glutamine (Thermo Fisher Scientific, Waltham, MA), supplemented with 10% fetal bovine serum (HyClone). *E*. *chaffeensis* (Arkansas strain) was cultivated in THP-1 cells as previously described [77].

### Cell lysis and protein extraction

*E*. *chaffeensis*-infected THP-1 cells were harvested at 24, 48 and 72 h post infection (hpi) and whole-cell lysates (uninfected THP-1 cells were used as controls) were extracted three times in complete-RIPA buffer supplemented with cOmplete Protease Inhibitor Cocktail tablet (Sigma-Aldrich, St. Louis, MO) and Halt Phosphatase Inhibitor Cocktail (Thermo Fisher Scientific). Lysates were centrifuged at 13,000 x *g* for 30 s to pellet insoluble material and cleared by centrifugation at 13,000 x *g* for 20 min at 4°C. The protein concentration was determined using Pierce BCA Protein Assay (Thermo Fisher Scientific). In addition, equal mass amount of *N*-ethylmaleimide (NEM; Thermo Fisher Scientific) was added to the whole cell lysates to preserve native ubiquitination of proteins in co-immunoprecipitation experiment performed for Fig 4A. Lastly, for the western blot experiment in Fig 4B, 26S proteasome inhibitor, bortezomib (Thermo Fisher Scientific), was added to cell culture at 10ng/ml concentration for 10 h before whole cell lysates were collected.

### RNA extraction, cDNA synthesis and qRT-PCR

Total RNA from *E*. *chaffeensis*-infected and uninfected THP-1 cells was isolated with the RNeasy Mini Kit (Qiagen, Beverly, MA) using on-column DNA digestion with RNase-free DNase reagent (Qiagen). cDNA synthesis was performed with total RNA (1 μg) using qScript cDNA Synthesis Kit (Quantabio, Beverly, MA). Quantitative real-time PCR was performed with iQ SYBR Green SuperMix (Agilent Technologies, Santa Clara, CA) with gene-specific primers. Forward FBW7: 5’-CCACTGGGCTTGTACCATGTT-3’; reverse FBW7: 5’-CAGATGTAATTCGGCGTCGTT-3’. Forward GAPDH: 5’-GCTCTCTGCTCCTCCTGTTC-3’; reverse GAPDH: 5’-TTCCCGTTCTAGCCTTGAC-3’.

### RNAi and quantification of *E*. *chaffeensis* by qPCR

Specific FBW7 siRNAs were siGENOME SMARTpool siRNA (Dharmacon, Lafayette, CO), which are endoribonuclease-prepared siRNA pools containing heterogenous mixture of four different siRNAs targeting the same human FBW7 mRNA sequence. The control siRNA was ON-TARGET*plus* non-targeting siRNA (Dharmacon) designed to have fewer off-targets than standard unmodified negative control siRNAs. Quantification of *E*. *chaffeensis* by qPCR after RNA interference has been previously described [27]. Fold changes shown were calculated by first normalizing transfected FBW7 siRNA to cells transfected with scrambled siRNA (infected and uninfected, respectively). Then, the infected group was normalized to uninfected group to finally obtain the fold change.

### Confocal immunofluorescent microscopy

THP-1 cells were plated in 6-well plates and infected with cell-free *E*. *chaffeensis* at a multiplicity of infection (MOI) of 100 for 24, 48 and 72 h. Cells were cytocentrifuged onto glass slides and fixed in ice-cold 4% paraformaldehyde in PBS for 20 min then permeabilized and blocked in 1% Triton-X 100 in PBS with 5% bovine serum albumin for 1 h. The cells were incubated with TRP120 rabbit peptide antisera (1:1000) and anti-FBW7 mouse monoclonal antibody (1:100; R&D Systems, Minneapolis, MN) for 1 h, washed three times with PBS and incubated with anti-rabbit Alexa Fluor 488-IgG (H+L) and anti-mouse Alexa Fluor 594-IgG (H+L) secondary antibodies for 1 h. Slides were washed three times with PBS and mounted with ProLong Gold Antifade reagent with 4’,6-diamidino-2-phenylindole (DAPI; Invitrogen). Images were obtained using a Zeiss Laser Scanning Microscopy 880 with Airyscan and processed using Zeiss ZEN Microscopy Software (ZEISS, Oberkochen, Germany) and FIJI (FIJI Is Just ImageJ).

### Generation of FBW7 and TRP120 constructs, and recombinant TRP120

FBW7 truncation constructs (FBW7-N, FBW7-FBOX, and FBW7-WD40) were created using PCR amplification from pGEM-FBW7 plasmid (Sino Biological, Wayne, PA) and cloning into the pcDNA3.1/His mammalian expression vector (Thermo Fisher Scientific). Plasmid DNA of FBW7-N, FBW7-FBOX and FBW7-WD40 clones was obtained from transformed TOP10 *E*. *coli* cultures and purified using QIAprep Spin Maxiprep kit (Qiagen). Full-length FBW7 plasmid was purchased as pcDNA3.1+/C-(K) DYK-FBW7 (GenScript, Piscataway, NJ). The same FBW7 domain constructs were also used for purification from HeLa cells through immunoprecipitation to obtain recombinant proteins used in co-IP experiment (S1 Fig). Full length and tandem repeat-C-terminal domain (TR-C) and mutant plasmids with cysteine to serine point mutations at the C-terminus (C520S) of TRP120 were gene synthesized and cloned into pcDNA3.1+C-eGFP (GenScript). Recombinant TRP120-His used for *in vitro* ubiquitination experiments was purified from TOP10 *E*. *coli* transformed with pBAD/TOPO-Thio-TRP120 plasmid as previously described [7,9]. Recombinant TRP120-TR-GST was used for co-IP experiment (S1 Fig) was purified using glutathione Sepharose 4B (GE) from *E*. *coli* BL21 cells transformed with pGEX-6p1-TRP120-TR plasmid.

### Transfection and immunofluorescent microscopy

The plasmid DNA was transfected into HeLa cells using Lipofectamine 2000 (Invitrogen) according to manufacturer’s protocol. Cells ectopically expressing TRP120 or FBW7-His were cytocentrifuged onto glass slides and fixed in ice-cold 4% paraformaldehyde in PBS for 20 min then permeabilized and blocked in 1% Triton-X 100 in PBS with 5% bovine serum albumin for 1 h. The cells were incubated with rabbit anti-TRP120 (1:1000) and mouse anti-His antibodies (1:100) at room temperature for 1 h, then washed and incubated with anti-rabbit Alexa Fluor 488-IgG (H+L) and anti-mouse Alexa Fluor 594-IgG (H+L) secondary antibodies for 1 h. Images were obtained using an Olympus BX61 epifluorescence microscope and analyzed using SlideBook 6 Reader software (Intelligent Imaging Innovations, Denver, CO) and FIJI.

### Quantitative microscopy and Mander’s overlap coefficient (MOC)

Quantification of immunofluorescence microscopy and calculation of Mander’s coefficient were carried out using WCIF-ImageJ (Bob and Joan Wright Cell Imaging Facility, Krembil Research Institute), which contains plugin for intensity correlation analysis (ICA) and corrected total cell fluorescence (CTCF). ICA was used to calculate Mander’s overlap coefficient (MOC) by using the selection tool to mark the regions of interest. Similarly, CTCF values were collected using the measurement tool after the selection of the regions of interest in a micrograph.

### *In vitro* precipitation of recombinant TRP120 and FBW7

Recombinant TRP120-GST was incubated at 4°C overnight with recombinant FBW7-His or recombinant WD40-His in native equilibration buffer (50 mM NaH2PO4, 300mM NaCl, pH = 7.0). All proteins were also incubated alone as control. Samples were placed onto cOmplete His-Tag Purification Resin (Sigma-Aldrich) to immobilize the His-tagged bait from the samples overnight at 4°C. Unbound protein was washed away with equilibration buffer containing 5mM, 10mM, or 20mM Imidazole. Protein-protein interaction complexes were eluted using equilibration buffer containing 500mM Imidazole. Western blot analysis was performed on eluted samples. Detection of FBW7 and WD40 was performed with anti-His antibody and TRP120 was detected using both anti-TRP120 or anti-GST antibody.

### Co-Immunoprecipitation and Western immunoblot

Co-Immunoprecipitation (co-IP) was performed with 3-day post-infection (95–100%) THP-1 cells using Pierce Protein A/G Agarose kit (Thermo Fisher Scientific) according to manufacturer’s protocol. Protein samples were resolved by SDS-PAGE, transferred onto nitrocellulose membranes, and blocked for 1 h at room temperature in Tris-buffered saline with 5% nonfat dry milk and 1% Triton-X 100. Primary antibodies included mouse anti-FBW7 (1:1000; R&D Systems, used to detect both unmodified and ubiquitinated FBW7), rabbit anti-TRP120 peptide antisera (1:10,000), rabbit anti-GAPDH (1:10,000; Proteintech, Rosemont, IL), rabbit anti-NICD (1:1000; Cell Signaling Technology, Danvers, MA), rabbit anti-p-c-Jun (1:1000; Cell Signaling Technology), rabbit anti-MCL1 (1:1000; Cell Signaling Technology), rabbit anti-K48 linkage-HRP conjugated (1:1000; Cell Signaling Technology), mouse anti-FK2 (1:500; Cell Signaling Technology), and mouse anti-cMYC (9E10) (1:500, Santa Cruz Biotechnology, Dallas, TX). Secondary antibodies included horseradish peroxidase-labeled goat anti-rabbit IgG and anti-mouse IgG (1:20,000; Kirkegaard & Perry, Gaithersburg, MD). Densitometry was performed using ImageJ software.

### *In vitro* FBW7 ubiquitination assay

All *in vitro* ubiquitination assays were performed with a Ubiquitinylation kit (Enzo Life Sciences, Farmingdale, NY). FBW7 ubiquitination was performed using purified recombinant TRP120 and native or recombinant FBW7. FBW7 (100 nM) was added to a ubiquitination reaction containing Ub, ATP, inorganic pyrophosphatase and Mg^2+^ (buffer) in presence of E1, UbcH5b E2 and TRP120 (10 nM) as the E3. Negative control reaction did not contain ATP. The assay was performed according to manufacturer’s protocol. Ubiquitination reaction was performed at 37°C for 4 h, and the reaction was stopped with the addition of Laemmli buffer. The samples were boiled for 5 min and resolved by SDS-PAGE for Western blot analysis using anti-FBW7, anti-TRP120, and anti-K48 antibodies.

### Statistical analysis

The results were evaluated using two-tailed Student *t*-test with *p*-values of ≤0.05 considered statistically significant.

## Supporting information

S1 Fig Direct interaction between TRP120-TR with FBW7 domains. *In vitro* pull-down was performed to demonstrate direct interaction between recombinant *E*. *chaffeensis* TRP120 tandem-repeat (TR) domain protein with recombinant FBW7 FBOX and WD40 domains. cOmplete His-Tag purification resin was used to pull down FBOX-His and WD40-His proteins, and bound TRP120-TR-GST was detected with anti-GST and anti-TRP120 antibodies.
(TIF)

S2 Fig Western immunoblots to demonstrate protein stability of FBW7 FBOX and WD40 domains. His-tagged FBW7 domain constructs were transfected into HeLa cells and whole cell lysates were obtained at 48 hours post-transfection (hpt). Western immunoblots were performed to demonstrate stability of ectopically expressed FBW7 domain constructs detected by anti-His antibody.
(TIF)
