# Supplementary figures and images for: Ehrlichia chaffeensis TRP120-mediated ubiquitination and proteasomal degradation of tumor suppressor FBW7 increases oncoprotein stability and promotes infection
Source: PLoS Pathog. 2020 Apr 30;16(4):e1008541. doi: 10.1371/journal.ppat.1008541 (PMC7217479; doi:10.1371/journal.ppat.1008541)

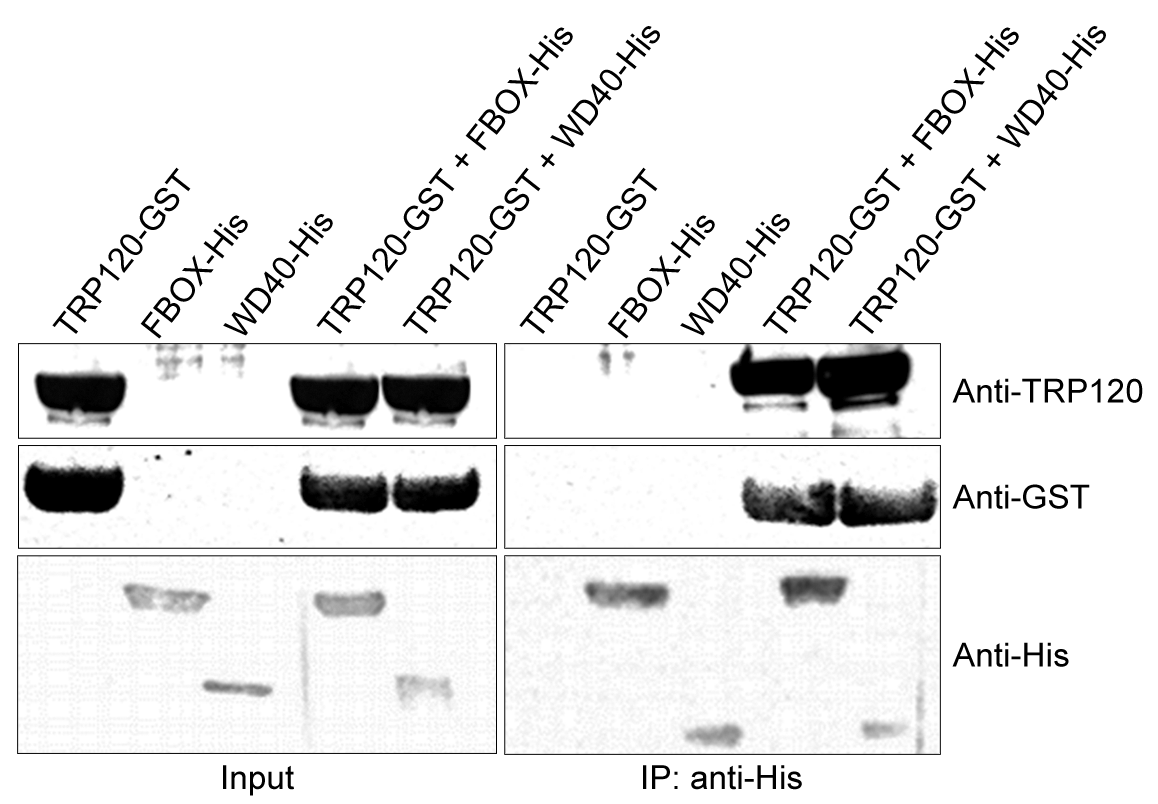

Supplement: S1 Fig — In vitro pull-down was performed to demonstrate direct interaction between recombinant E. chaffeensis TRP120 tandem-repeat (TR) domain protein with recombinant FBW7 FBOX and WD40 domains. cOmplete His-Tag purification resin was used to pull down FBOX-His and WD40-His proteins, and bound TRP120-TR-GST was detected with anti-GST and anti-TRP120 antibodies. (TIF) [file ppat.1008541.s001.tif]

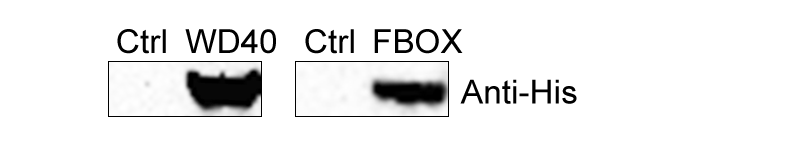

Supplement: S2 Fig — His-tagged FBW7 domain constructs were transfected into HeLa cells and whole cell lysates were obtained at 48 hours post-transfection (hpt). Western immunoblots were performed to demonstrate stability of ectopically expressed FBW7 domain constructs detected by anti-His antibody. (TIF) [file ppat.1008541.s002.tif]
